# Diurnal and Seasonal Change in Stem Respiration of *Larix principis-rupprechtii* Trees, Northern China

**DOI:** 10.1371/journal.pone.0089294

**Published:** 2014-02-26

**Authors:** Yan Yang, Miao Zhao, Xiangtao Xu, Zhenzhong Sun, Guodong Yin, Shilong Piao

**Affiliations:** 1 Department of Ecology, College of Urban and Environmental Sciences, and Key Laboratory for Earth Surface Processes of the Ministry of Education, Peking University, Beijing, China; 2 Department of Geosciences, Princeton University, Princeton, New Jersey, United States of America; Tennessee State University, United States of America

## Abstract

Stem respiration is a critical and uncertain component of ecosystem carbon cycle. Few studies reported diurnal change in stem respiration as well as its linkage with climate. In this study, we investigated the diurnal and seasonal change in stem respiration and its linkage with environmental factors, in larch plantations of northern China from 2010 to 2012. The stem respiration per unit surface area (R_S_) showed clear diurnal cycles, ranging from 1.65±0.10 to 2.69±0.15 µmol m^−2^ s^−1^, increased after 6∶00, peaked at 15∶00 and then decreased. Both stem temperature and air temperature show similar diurnal pattern, while the diurnal pattern of air relative humidity is just the opposite to Rs. Similar to the diurnal cycles, seasonal change in R_S_ followed the pattern of stem temperature. R_S_ increased from May (1.28±0.07 µmol m^−2^ s^−1^) when the stem temperature was relatively low and peaked in July (3.02±0.10 µmol m^−2^ s^−1^) when the stem temperature was also the highest. Further regression analyses show that R_S_ exponentially increases with increasing temperature, and the Q_10_ of Rs at mid daytime (1.97±0.17 at 12∶00 and 1.96±0.10 at 15∶00) is significantly lower than that of mid nighttime (2.60±0.14 at 00∶00 and 2.71±0.25 at 03∶00) Q_10_. This result not only implies that Rs is more sensitive to night than day warming, but also highlights that temperature responses of Rs estimated by only daytime measurement can lead to underestimated stem respiration increase under global warming, especially considering that temperature increase is faster during nighttime.

## Introduction

Rising atmospheric carbon dioxide (CO_2_) is considered to have significant impacts on the climate system [1], which has triggered strong scientific interests in understanding the global carbon cycle. Forests play a key role in the global carbon cycle. They cover approximately one-third of the earth’s land surface, and store about 861±66 Pg of the total carbon [2]. Current terrestrial carbon sink has also been suggested to be mainly contributed by the forest sink [2]. Accordingly, accurate information on processes related to forest carbon cycle is essential to predict future evolution of the global carbon cycle and climate change. As a major pathway of carbon loss from terrestrial ecosystems, ecosystem respiration is critical to regulating forest ecosystem carbon fluxes and thus important to forest carbon balance. Ecosystem respiration is composed of two dominant fluxes, (i) soil respiration including heterotrophic respiration of decomposing microbes, respiration of plant roots and soil fauna, and (ii) above-ground respiration of plant woody tissues and leaves. Compared with soil respiration [3]–[5], our understanding of the linkage between above-ground respiration and climate is very limited [6], [7].

As an important part of woody tissues, stem respiration contributes 9% of the total ecosystem respiration in boreal forest [8], [9], 9% in dry Mediterranean forests [10], about 14% in Neotropical rainforests [11] and up to 21% in temperate forests [12]. Both environmental and biotic factors can influence stem respiration [13]–[20]. Among them, temperature is well known to be a dominant environmental driver [6], [9], [21]–[23], and is often used to predict stem respiration [24]–[26]. Therefore, it is critical to accurately quantifying the temperature sensitivity of stem respiration, which may reduce the uncertainties in assessing the positive feedbacks between the carbon cycle and climate predicted by coupled carbon-climate models [27], [28].

Temperature sensitivity of stem respiration is usually expressed in terms of Q_10_ (the rate of change in respiration resulting from a 10°C increase in temperature). Numerous studies on temperature sensitivity of stem respiration have been conducted across different forest types of the world and reported different Q_10_ values of stem respiration for different forests, varying from 1.00 to 6.40 [26], [29]–[32]. It should be noted, however, that most of these previous studies estimated Q_10_ values based on the measurement of daytime stem respiration [33], and few studies measured diurnal change in stem respiration as well as its linkage with climate [34]. Since stem respiration is also influenced by other environmental and physiological processes [16], [23], [35]–[39], such as photosynthesis that occurs only during the daytime, it is possible that stem respiration responds to temperature changes in daytime and nighttime differently. Furthermore, both observations and model projection have showed that global warming is faster during the nighttime than that during the daytime [1]. Thus, understanding the possible differential responses of stem respiration to day and night warming will be helpful to improve the projection of future carbon cycle evolution as well as its feedback to climate.

In this study, we have conducted field measurement to investigate the diurnal and seasonal change in stem respiration and its linkage with environmental factors, in larch plantations of northern China since 2010. The primary object of this paper is to test the hypothesis that temperature sensitivity of stem respiration is different during daytime and nighttime.

## Materials and Methods

### Study Site and Experimental Design

**Figure 1:**
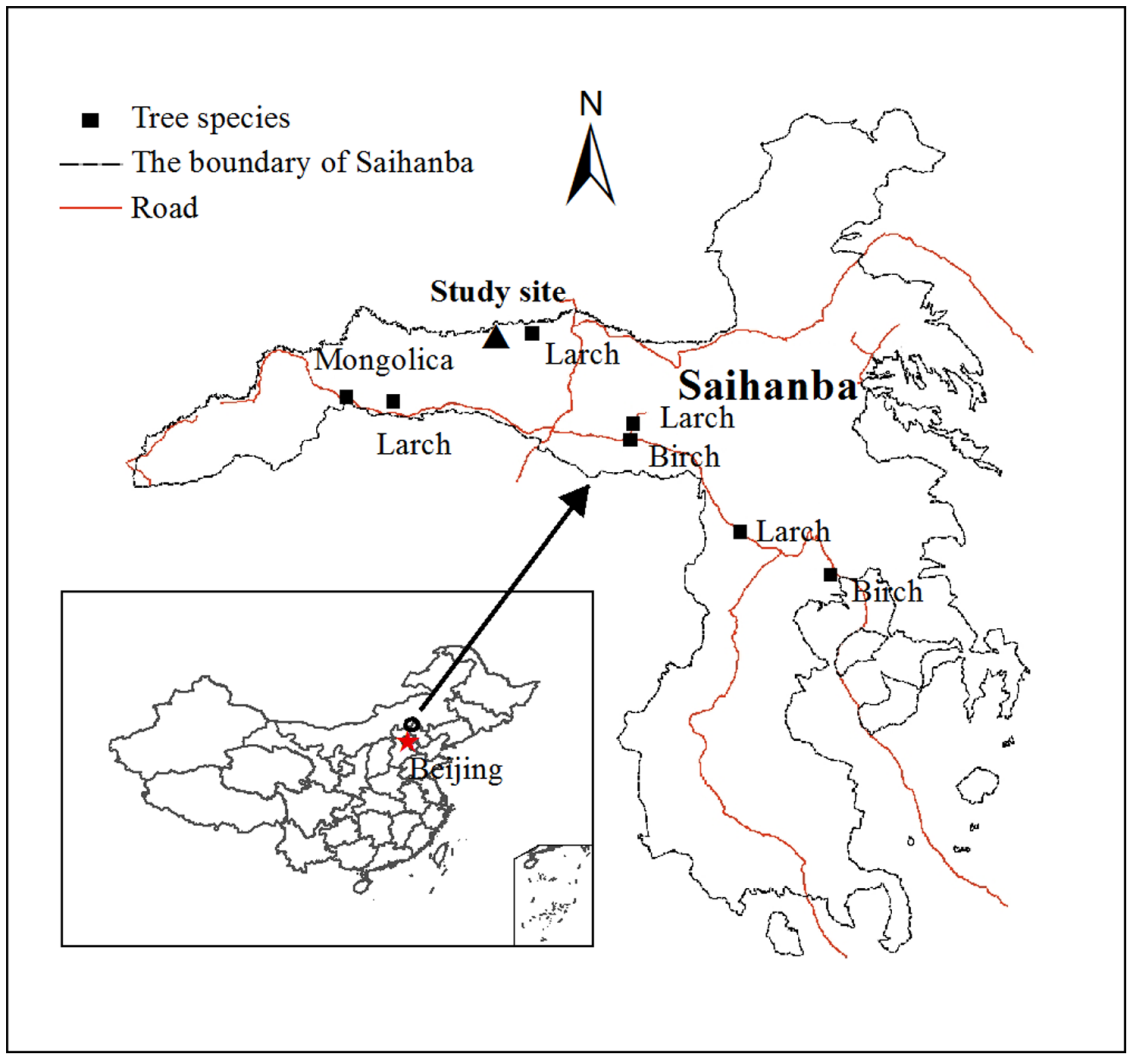
Location of study site in Saihanba National Forest Park, Hebei Province, China.

This study was conducted at Saihanba ecological station (42°24.723′N, 117°14.844′E, 1505 m a.s.l) of Peking University, situated in Saihanba National Forest Park, Hebei Province (Fig. 1). Saihanba has a mean annual precipitation of approximately 450 mm, 70% of which occurs from June to August, and mean annual temperature of −1.4°C [40] with a long cold winter and a short growing season (May-September). The soils are predominantly sand. Soil bulk density is 1.47 g cm^−3^, C:N ratio is 8.9±0.3, and soil pH (soil:water, 1∶2.5) is 6.3±0.2 [41].

The experiment was carried out in three 20×20 m plots located within a 45-year-old larch plantation (*Larix principis-rupprechtii*). The topography of the plots is nearly flat and the stem density is 870±48 stem·ha^−1^ with an average diameter at breast height (DBH) of 19.9±2.8 cm and an average height of 15.8±1.6 m. Two larch trees were chosen randomly in each plot, and all together 6 trees were selected in the study area with an average DBH of 20±2 cm and an average height of 16±1.5 m. Although the experiment plots and individuals were very homogeneous, it should be reminded that the limited sample size (6 trees in total) might introduce biases originated from inter-individual differences and the effect of micro-topography. In this study, 3-hourly stem respiration measurements were made for a whole day, twice a month in growing season from 2010 to 2012, using a LI-6400-09 (Li-Cor, Lincoln, Nebraska, USA). In order to capture the CO_2_ released by stems, a technique called horizontally oriented soil chamber (HOSC) [12], [42] was exploited: the CO_2_ chamber (9.9 cm in diameter) was connected to stem collars (10.1 cm in diameter), which completely enclosed a 10.1 cm segment of the tree stem at 1.3 m above ground and were fixed tightly onto the stems with nylon straps. To ensure an airtight seal between stem collars and stem surfaces, loose barks at two ends of the enclosed stem segment, which might leak air, were removed at first. Then the collars made of polyvinyl chloride (PVC) pipe were polished to fit the curvature of the stem surface. Finally the small gaps between the collars and stem surfaces were sealed completely with silicon sealant. Meanwhile, stem temperatures were measured with copper-constantan thermocouples at the depth of 5 mm from the stem surface with tree bark.

### Data Analyses

To eliminate the influence of plant size, the measured stem respiration was firstly normalized by the surface area enclosed, which was calculated using the following equation [43]

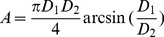
(1)where *A* is the stem surface area enclosed by the collar (m^2^), *D_1_* is the diameter of the chamber (m), *D_2_* is the diameter of the enclosed stem segment (m). Then the stem respiration per unit surface area R_S_ (µmol·m^−2^·s^−1^) should be the measurement results divided by the area *A* for each plant.

The relationship between stem respiration and corresponding stem temperature can be described by an exponential function
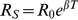
(2)where T is the measured stem temperature (°C); R_S_ is the stem respiration per unit surface area (µmol·m^−2^·s^−1^) at temperature T; R_0_ is the potential stem respiration rate at 0°C and β is a fitting parameter, which indicates the temperature sensitivity of respiration [44]. The temperature sensitivity is often expressed by Q_10_, which describes the proportional change in stem respiration rate for a 10°C increase in sapwood temperature). According to Eq.2, the Q_10_ values can be calculated as



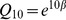
(3) For each plant individual, three-hourly R_0_ and β were acquired by fitting stem respiration (R) and stem temperature (T) measured throughout the sampling period to Eq.2 and three-hourly Q_10_ values were calculated according to Eq.3. Then they are averaged for all six larch individuals. All the statistical analyses were performed in PASW statistic 18 (SPSS Inc., Chicago, IL, USA).

## Results

### Diurnal Variation

**Figure 2:**
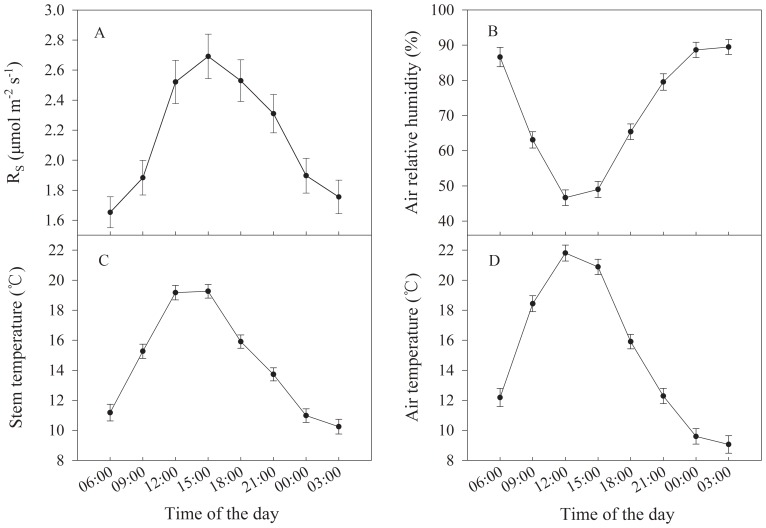
Diurnal changes in (A) stem respiration per unit surface area (RS), (B) air relative humidity, (C) stem temperature and (D) air temperature. For each 3-hourly interval, measurements were averaged for the six sample trees in the whole growing season (May to Sep) from 2010 to 2012. The resulting standard errors are represented by the bars.

The measured stem respiration per unit surface area (R_S_) and environmental factors showed clear diurnal cycles (Fig. 2), averaged over the whole sampling period. R_S_, ranging from 1.65±0.10 to 2.69±0.15 µmol m^−2^ s^−1^, increased after 6∶00, peaked at 15∶00 and then decreased. Both stem temperature and air temperature showed similar diurnal pattern. Nevertheless, stem temperature experienced a plateau after mid-day (12∶00–15∶00) and then decreased more quickly than R_S_. As shown in Fig. 2A and C, the stem temperature values were comparable between 09∶00 and 18∶00 but the R_S_ value was much larger at 18∶00. Air temperature had a similar fast afternoon decrease pattern as stem temperature did, but the amplitude of air temperature diurnal change (12.75°C) was larger than that of stem temperature (9.02°C). The diurnal pattern of air relative humidity was just the opposite to that of air temperature (Fig. 2B and D), ranging from 46.64±2.21% at 12∶00 to 89.45±2.13% at 3∶00.

### Seasonal Variation

**Figure 3:**
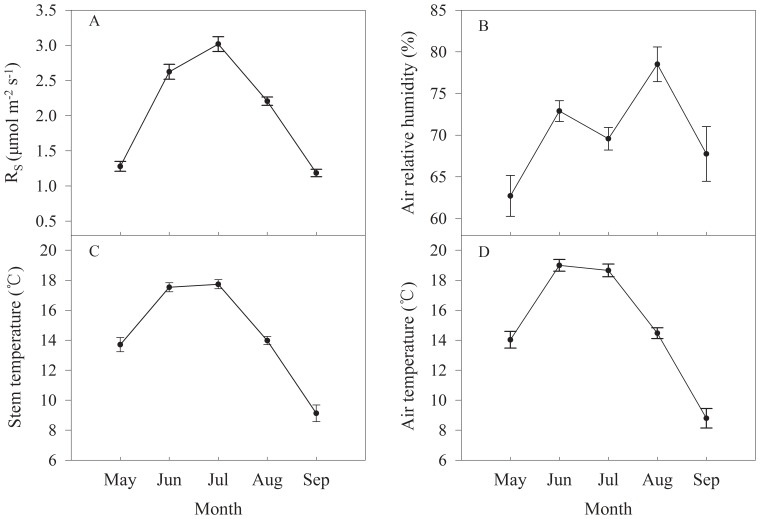
Seasonal changes in (A) stem respiration per unit surface area (R_S_), (B) air relative humidity, (C) stem temperature and (D) air temperature. For each month, all the 3-hourly measurements were averaged for the six sample trees from 2010 to 2012. The resulting standard errors are represented by the bars.

Figure 3 illustrated the seasonal changes of R_S_, air relative humidity, stem temperature, and air temperature from May to September. Similar to the diurnal cycles, R_S_ followed the pattern of stem temperature (Fig. 3A and C). R_S_ increased from May (1.28±0.07 µmol m^−2^ s^−1^) when the stem temperature was relatively low (13.70±0.47°C) and peaked in July (3.02±0.10 µmol m^−2^ s^−1^) when the stem temperature was also the highest (17.73±0.30°C). Both of them decreased afterwards to the lowest point in September, with R_S_ as 1.19±0.05 µmol m^−2^ s^−1^ and stem temperature as 9.13±0.56°C. It is also noteworthy that the R_S_ values did not differ very much between the start and the end of the growing season, while there was a significant gap between the stem temperature values (near 14°C in May but around 9°C in September). Similarly, the air temperature reached its peak value in June and July (Fig. 3D), and the minimum value occurred in September. Consistent with diurnal changes, seasonal maximum air temperatures were higher than maximum stem temperatures in June and July, meanwhile, seasonal minimum stem temperature was 8.80°C, lower than that of air temperature (9.13°C) in September. The seasonal pattern of air relative humidity was no longer the opposite to that of air temperature (Fig. 3B), which increased in early growing season, decreased a little in July, peaked in August and then dropped in September.

### Diurnal Change in Q_10_

**Figure 4:**
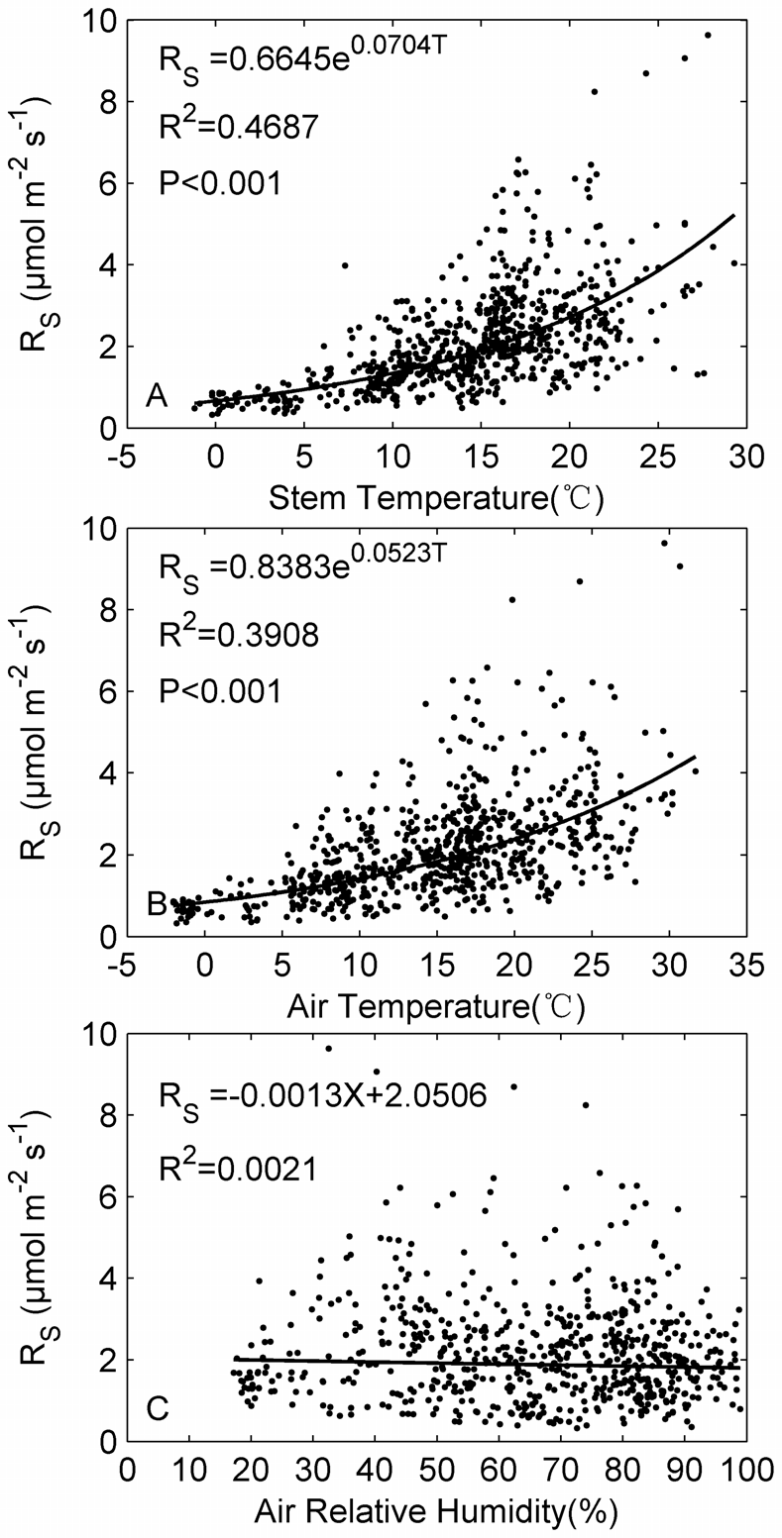
Relationship of the stem respiration per unit surface area (R_S_) with (A) stem temperature, (B) air temperature and (C) air relative humidity.

To gain further understanding of how environmental factors influence stem respiration activity, the stem respiration rates and temperature measured in the sampling period were fitted to Eq.2 (Fig. 4A and B) and stem respiration rates were also linearly regressed against the air relatively humidity (Fig. 4C). In general, R_S_ showed a good exponential relationship with both stem temperature (Fig. 4A, R ^2^ = 0.47, P<0.001) and air temperature (Fig. 4B, R ^2^ = 0.39, P<0.001). There was no good linear relationship between R_S_ and air relative humidity (Fig. 4C, R ^2^ = 0.00).

**Figure 5:**
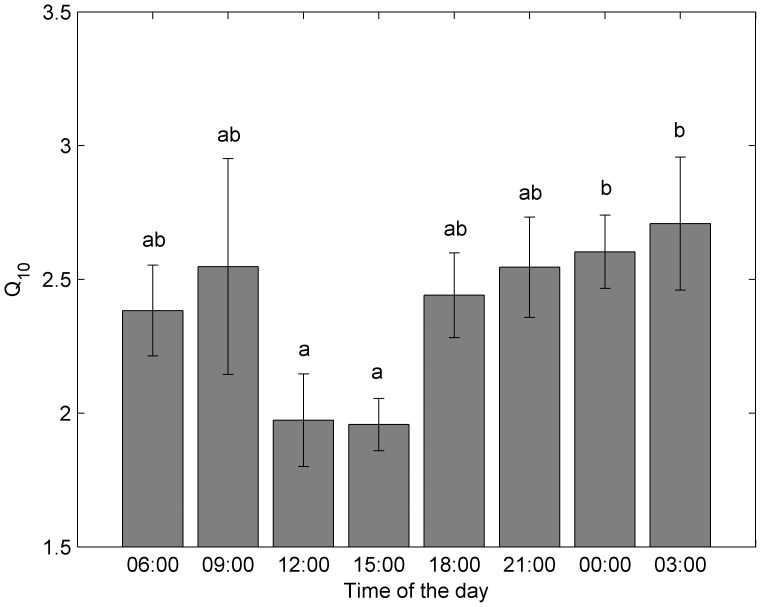
The diurnal variations of Q_10_ estimated based on the seasonal variation in R_S_ at different time during one day. Values not given a common letter are significantly different from each other at P = 0.05. Bars represent the standard error.

In order to investigate diurnal variations of temperate sensitivity of R_S_, Q_10_ values were further calculated based on the seasonal variation in R_S_ for each time during one day. There was statistically significant difference between daytime (1.97±0.17 at 12∶00 and 1.96±0.10 at 15∶00) and nighttime (2.60±0.14 at 00∶00 and 2.71±0.25 at 03∶00) Q_10_ (Fig. 5). Q_10_ values in other time intervals fell in between and were not significantly different from each other.

## Discussion

The magnitude of our R_S_ values (0.33–6.59 µmol m^−2^ s^−1^) is similar with previous studies on mature conifer forests. For example, Wang et al. [43] found that stem respiration rates in a 33-year-old larch forest varied from approximately 0.9 µmol m^−2^ s^−1^ to 6.6 µmol m^−2 ^s^−1^ in June, 2001 and Acosta et al. [8] documented that the R_S_ range of a 22-year-old Norway spruce forest stand during the growing season from 1999 to 2002 was 0.34–6.52 µmol m^−2^ s^−1^. Meanwhile, the mean stem respiration (2.15 µmol m^−2^ s^−1^) was lower than that of soil respiration rate (3.22 µmol m^−2^ s^−1^) [41] at the same plot. Bolstad et al. [45] showed that the stem respiration was lower than the soil respiration which was typically more than 60% of total ecosystem respiration during the growing season while Clinton et al. [46] showed that the mean stem CO_2_ efflux (2.60±0.17 µmol m^−1^ s^−2^) was slightly higher than that of soil CO_2_ efflux (2.53±0.11 µmol m^−1^ s^−2^).

Stem respiration rates can respond to temperature changes and plant activities like photosynthesis, plant growth, etc. [47], [48]. Our results show that variations of stem respiration rates in larch forests were largely influenced by diurnal and seasonal changes of stem temperature. During the study period, the maximum of the stem respiration occurred in the afternoon while the minimum occurred in the early morning within one day and stem respiration rates peaked in July in growing season, which is consistent with previous studies [9], [18], [23], [31], [42], [49]. For example, Zha et al. [9] found that stem respiration of Scots pine peaked at around 16 h and was highest in July. Acosta et al. [8] indicated that stem respiration of Norway spruce reached maximum between 13 h and 16 h and the highest rate occurred in June and July. Zhu et al. [23] suggested that stem respiration of Schima superba also followed a similar diurnal pattern, reaching the highest in the afternoon and the lowest at about 8∶00 in the early morning.

Nevertheless, stem temperature can’t fully explain all of the variations of R_S_ [9]. In our study, the R_S_ values of afternoon (18∶00) and late growing season (Aug) were higher than those in the morning (09∶00) and early growing season (May) while the stem temperature were comparable (Fig. 2A and C, Fig. 3A and C). This phenomenon suggests that plant activities like photosynthesis and cambium activity probably play an important role in regulating stem respiration changes. Martin et al. [50] found that when temperature and transpiration are constant, R_S_ appears to be positively correlated with substrate supply. The diurnal change of respiratory substrate, supplied by photosynthesis, may also influence the respiration rates [48], [51]. At seasonal scale, plant growth activities can’t be ignored. The stem respiration mainly consists of maintenance respiration and growth respiration [52]. Maintenance respiration varies primarily with changes in temperature and is also reported to increase with relative growth rate [37], [53]. Meanwhile, growth respiration is controlled by the timing and magnitude of plant growth [54]. That is to say, the stem respiration varies throughout the growing season, following not only the change of temperature, but also the change of phenology and environmental factors that control growth.

Accurate understanding of temperature response of respiration is critical in estimating global carbon balance and its response to current climate change. Our results show that Q_10_ values of Rs vary from 1.96 to 2.71, which are within the range reported by previous studies [9], [17], [31], [34], [42], [55], [56]. In growing season average diurnal cycle, Q_10_ values were lowest in mid-day (12∶00–15∶00), which may be partly explained by the acclimation of respiration to rising temperature. Both theory and observations have suggested a decline temperature sensitivity of rates of respiratory CO_2_ efflux from plants [52], [57], [58] and soils [5], [59]. For example, Tjoelker et al. [57] reported that Q_10_ value of foliar respiration decline by 0.04 in response to 1°C increase in mean ambient temperature. In deed, highest stem temperature is observed during the mid-day. Another possible explanation of the suppressed temperature sensitivity of stem respiration in mid-day (12∶00–15∶00) may be midday depression of photosynthesis, particularly during summer with stem temperature approaching 30°C and air temperature above 30°C (Fig. 4A&B). High midday temperature is considered to be able to induce stomata closure and photosynthesis depression in water-limited regions by both observations (pine forest in Canary Islands) [60] and theoretical models. Reduced stomatal conductance and photosynthesis rates in midday during summer may further decline Rs, and thus influencing Q_10_ values derived from seasonal variation of R_S_. Often in models [14], [31], [61], Q_10_ is set to be a constant value of 2, similar to the midday values and lower than the nighttime values from our study. This can lead to underestimated stem respiration increase under global warming, especially considering that temperature increase is faster during nighttime [1].

Vegetation activities have been shown to respond negatively to nighttime temperature increase in cold and mesic regions [65], probably due to increased carbon loss through respiration. Combined with stronger nighttime warming [1], [62], [63], our results imply that the carbon loss through respiration might increase more than former model projections [14], [31], [61], and might further cancel out the increased photosynthesis driven by daytime warming in those areas. In contrast, ecosystems in arid and semi-arid regions are thought to respond to night time temperature change in a more complex way [65]. A manipulative experiment study in a temperate steppe ecosystem in north China reported that daytime warming induced reduction in gross ecosystem productivity (GEP), and night-time warming stimulated photosynthesis and GEP in the following day because enhanced respiration drew down the leaf carbohydrates concentration [64]. With higher stem respiration sensitivity at night, the stimulation effect might be strengthened in the future, while it is also possible that the carbon loss through enhanced nighttime respiration goes up even faster and cancels out the stimulating effect. Thus, more experimental researches as well as modelling efforts are necessary to accurately quantify the temperature sensitivity of stem respiration and to better address its implications on future vegetation dynamics.

## Conclusion

Temperature responses of rates of respiratory CO_2_ efflux from plants and soils are generally modelled using exponential functions with a constant Q_10_ near 2.0, similar to the midday values and lower than the nighttime values from our study. This result has important implications for the predictions of forest responses to warming. Current carbon cycle models must consider diurnal change in temperature sensitivity of Rs to accurately predict ecosystem C cycling under climate warming. In the future, additional experiments with larger sample size need to be performed in other ecosystems in order to draw a more generalized conclusion and to further address detailed mechanisms responsible for diurnal change in Q_10_ of Rs.
